# Supplementary material for: The Small RNA Universe of Capitella teleta
Source: Front Mol Biosci. 2022 Feb 25;9:802814. doi: 10.3389/fmolb.2022.802814 (PMC8915122; doi:10.3389/fmolb.2022.802814)
Supplement: Supplementary file 1 [file DataSheet1.ZIP › Supplement/confident/CAPTEscaffold_324_18356.pdf]

Provisional ID	:	CAPTEscaffold_324_18356
Score total	:	136
Score for star read(s)	:	3.9
Score for read counts	:	130.1
Score for mfe	:	1
Score for randfold	:	1.6
Score for cons. seed	:	-0.6
Total read count	:	267
Mature read count	:	221
Loop read count	:	0
Star read count	:	46

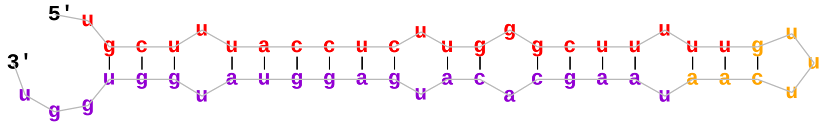

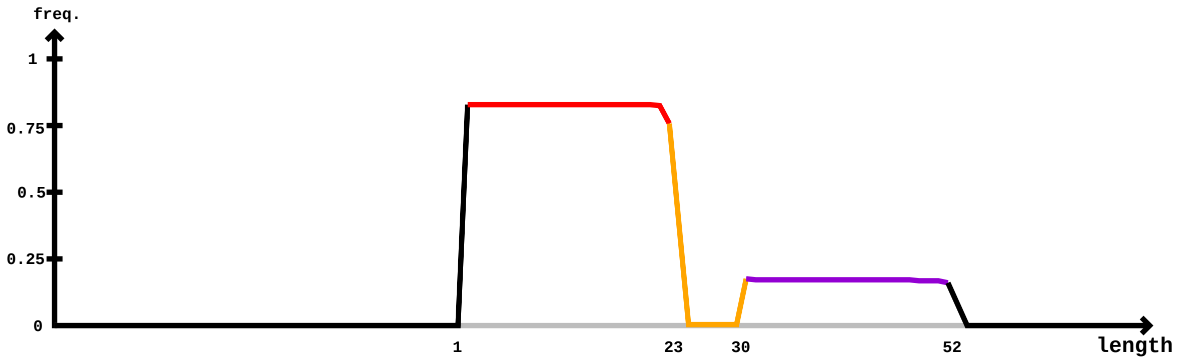

Mature Star

```
5'- gcaauugacccagucaucuaacuauucaucucggcuuguguugcuuuaccucuugggcuuuuuguuucaauaagcacaugagguaugguggugugugcacaagucuuugcaa   -3'    obs
    gcaauugacccagucaucuaacuauucaucucggcuuguguugcuuuaccucuugggcuuuuuguuucaauaagcacaugagguaugguggugugugcacaagucuuugcaa          exp
    ((((.((((...))))................(((((((((((((.((((((.((.((((.(((...))).)))).)).)))))).))))......)))))))))).))))..   reads      mm         sample
    .........................................ugcuuuaccucuugggcuuu...................................................   1          0          seq
    .........................................ugcuuuaccucuugggcuuuu..................................................   18         0          seq
    .........................................ugcuuAaccucuugggcuuuuu.................................................   1          1          seq
    .........................................ugcuuuaccucuugggUuuuuu.................................................   2          1          seq
    .........................................ugcuuuaccucuuAggcuuuuu.................................................   2          1          seq
    .........................................ugcuuuaccucuugggcuuuuu.................................................   150        0          seq
    .........................................ugcuuuaccucuugggcuGuuu.................................................   12         1          seq
    .........................................ugcuuuaccucuugggcuuuuuU................................................   30         1          seq
    .........................................ugcuuuaccucuugggcuuuuuA................................................   5          1          seq
    .........................................ugcuuuaccucuugggcuuuuuguuucaau.........................................   1          0          seq
    ......................................................................uaagcacaugagguaugg........................   1          0          seq
    ......................................................................uaagcacaugagguauggugg.....................   2          0          seq
    ......................................................................uaagcacaugagguaugguggu....................   42         0          seq
    ......................................................................uaagcacGugagguaugguggu....................   1          1          seq
```
